# Next generation sequencing reveals the antibiotic resistant variants in the genome of *Pseudomonas aeruginosa*

**DOI:** 10.1371/journal.pone.0182524

**Published:** 2017-08-10

**Authors:** Babu Ramanathan, Hassan Mahmood Jindal, Cheng Foh Le, Ranganath Gudimella, Arif Anwar, Rozaimi Razali, Johan Poole-Johnson, Rishya Manikam, Shamala Devi Sekaran

**Affiliations:** 1 Department of Biological Sciences, School of Science and Technology, Sunway University, Kuala Lumpur, Malaysia; 2 Department of Medical Microbiology, Faculty of Medicine, University of Malaya, Kuala Lumpur, Malaysia; 3 School of Pharmacy, Faculty of Science, University of Nottingham Malaysia Campus, Jalan Broga, Selangor, Malaysia; 4 Sengenics, High Impact Research (HIR), University of Malaya, Kuala Lumpur, Malaysia; 5 Department of Trauma and Emergency, University Malaya Medical Centre, Kuala Lumpur, Malaysia; Institut Pasteur of Shanghai Chinese Academy of Sciences, CHINA

## Abstract

Rapid progress in next generation sequencing and allied computational tools have aided in identification of single nucleotide variants in genomes of several organisms. In the present study, we have investigated single nucleotide polymorphism (SNP) in ten multi-antibiotic resistant *Pseudomonas aeruginosa* clinical isolates. All the draft genomes were submitted to Rapid Annotations using Subsystems Technology (RAST) web server and the predicted protein sequences were used for comparison. Non-synonymous single nucleotide polymorphism (nsSNP) found in the clinical isolates compared to the reference genome (PAO1), and the comparison of nsSNPs between antibiotic resistant and susceptible clinical isolates revealed insights into the genome variation. These nsSNPs identified in the multi-drug resistant clinical isolates were found to be altering a single amino acid in several antibiotic resistant genes. We found mutations in genes encoding efflux pump systems, cell wall, DNA replication and genes involved in repair mechanism. In addition, nucleotide deletions in the genome and mutations leading to generation of stop codons were also observed in the antibiotic resistant clinical isolates. Next generation sequencing is a powerful tool to compare the whole genomes and analyse the single base pair variations found within the antibiotic resistant genes. We identified specific mutations within antibiotic resistant genes compared to the susceptible strain of the same bacterial species and these findings may provide insights to understand the role of single nucleotide variants in antibiotic resistance.

## Background

*Pseudomonas aeruginosa* is a major opportunistic pathogen causing acute and chronic infections in human community. It is a fascinating, ubiquitous, gram-negative bacterium that can thrive at low densities within the range of 4°C to 42°C and involves in a range of interactions with eukaryotic hosts [1, 2]. The infection leads to most serious manifestations such as bacteremia, pneumonia, urinary tract infections and wound infections [3]. *P*. *aeruginosa* is intrinsically resistant to many antibiotics and the challenge is selection of the most appropriate antibiotic as the organism can develop resistance during treatment [4, 5].

The infection caused by multi-drug resistant (MDR) *P*. *aeruginosa* often leads to morbidity, mortality, chronic care and an increase towards the overall cost of treating the infection [6, 7]. Mutational changes found within the resistant genes on plasmids or mutations that alter the function of genes encoded by chromosomes may collectively contribute to the antibiotic resistant mechanisms [8]. The antibiotic resistant mechanisms include the acquisition of extended spectrum β-lactamases, carbapenemases, aminoglycoside-modifying enzymes and 16S ribosomal ribonucleic acid (rRNA) methylases. Mutational changes causing the up regulation of multidrug efflux pumps, depression of ampC, modification of antimicrobial targets and changes in the outer membrane permeability barrier have also been reported [8].

Epidemiological typing of *P*. *aeruginosa* requires the identification of stable distinguishing characteristics. Current molecular epidemiological typing techniques in practice such as variable-number tandem repeat (VNTR) and pulsed-field gel electrophoresis (PFGE) have helped to reveal the outbreaks of *P*. *aeruginosa* in hospital settings [9, 10]. However, limited resolution, cost and complex workflow of these systems make the typing in reference laboratories be performed in batch mode that affects the real time sequencing efforts. In addition, the traditional typing methods lacks in providing insights into the evolutionary significance of pathogens [11]. Multilocus sequence typing (MLST) to identify specific genes in clinical isolates has also been reported to be an effective genotyping method [12].

Application of next-generation sequencing (NGS) technology for whole genome, whole proteome and whole transcriptome sequencing is becoming popular. NGS is quick and high throughput technique that follows a single unified workflow. Using this technique, it is possible to identify the single-base-pair mutations within same bacterial species that can replace the traditional molecular typing methods for bacterial pathogens [13]. This approach provides greater sequence resolution than traditional methods by delivering a definitive catalog of genetic polymorphisms, particularly single-nucleotide polymorphisms (SNPs). WGS also associates epidemiology to genome evolution, genome structure, pathogen biology and gene content; which provides insights to biological markers, such as antibiotic resistance and virulence factors [14].

Identification of SNPs in bacterial genomes play a key role to determine the relationship between antibiotic resistant clinical isolates and tracing their evolutionary counterpart [15, 16]. Several studies have reported the genetic variants in cystic fibrosis disease conditions where the pathogen has emerged because of mutation and long-term colonization [17, 18]. In addition, genome sequencing facilitates high throughput screening for specific mutations within antibiotic resistant genes compared to the susceptible and/or reference strain of the same bacterial species. Thus, in the present study, we examined single nucleotide variations within the antibiotic resistant and susceptible *P*. *aeruginosa* clinical isolates collected from patients admitted at the University Malaya Medical Centre, Kuala Lumpur, Malaysia. These nsSNPs found within the antibiotic resistant isolates could potentially alter the amino acid sequence and may affect the stability and/or the function of the resulting protein expression.

## Materials and methods

### Bacterial strains and culture condition

*P*. *aeruginosa* isolates were collected from different hospital wards (Table 1) at the University Malaya Medical Centre, Kuala Lumpur, Malaysia over a period of 1 year from April 2009 to March 2010. The clinical strains were isolated from urine, wound, blood and indwelling medical devices. Isolates were frozen with Luria Bertani (LB) broth containing 30% V/V glycerol and stored at -80°C until used. The protocol for this study was approved by the Medical Ethics Committee, University Malaya Medical Centre, Kuala Lumpur, Malaysia.

**Table 1:** Selected *P*. *aeruginosa* clinical isolates included in the study from University Malaya Medical Centre, Kuala Lumpur, Malaysia over a period of 1 year from April 2009 to March 2010.

Isolate	Ward	Specimen
PAS1	Surgery	Wound
PAS2	Orthopedic	Wound
PAS3	Surgery	Urine
PAS4	Surgery	Urine
PAS5	Surgery	Wound
PAS6	Medical	Urine
PAS7	Surgery	Urine
PAS8	Pediatric	Blood
PAS9	Pediatric	Wound
PAS10	Medical	Blood

### Antimicrobial susceptibility testing

The antimicrobial susceptibility of isolates was tested using the disc diffusion method (E-test**®**) against following nine different antibiotics; piperacillin/tazobactam (PPT), ceftazidime (CAZ), aztreonam (AZT), amikacin (AK), gentamicin (GN), ciprofloxacin (CIP), imipenem (IMP), meropenem (MPM) and colistin (CL). The reference strain *P*. *aeruginosa* ATCC # 27853 was used to validate the technique. The calibrated carrier strip and the intersection of the strip to the edge of the inhibitory zone indicates the minimum inhibitory concentration (MIC) [19]. The clinical isolates were chosen for whole genome sequencing based on the antimicrobial susceptibility levels screened against different antibiotics.

### High-throughput whole genome sequencing

Genomic DNA was isolated from *P*. *aeruginosa* clinical isolates using the DNeasy DNA extraction kit (Qiagen, USA) according to the manufacturer’s instructions. The samples were then quantified using Qubit. All genomic DNA were fragmented using Covaris S2 at the temperature of 5.5 to 6°C for 40 seconds. The fragmented DNA was end repaired, added with dA base and ligated with Illumina indexed adapters. Size selections of the samples were performed using Invitrogen 2% agarose E-gels. The selected DNA fragments with adapter molecules on both ends underwent 10 cycles of PCR amplification and was sequenced using whole genome shotgun Illumina Hiseq 2000 flow cell V3 with paired-end libraries (~200 base-pair insert size).

### Read pre-processing

Adapter sequences and low quality reads were removed with quality score filter of ≥ 30 using PRINSEQ version 0.20.3 [20]. The following types of reads were removed: reads having ‘N’ in more than 10% of the total bases of that read, reads with Phred quality score less than 20 and reads shorter than 50 bp.

### Genome assembly

The sequenced reads were assembled using SPAdes Assembler version 3.8.1 [21]. Assembler was run using iterative kmer lengths ranging from 27 to 77. The assemblies were mapped against *P*. *aeruginosa* reference genome POA1 to evaluate the core genome average identities and completeness. The removal of probable contaminants was performed by BLAST against the assembly sequences of mitochondrial and primates’/rodents chromosome databases. The finalized draft genomes were submitted to Rapid Automated using Subsystem Technology (RAST) [22] server for gene predictions and annotations. Prokka Version 1.11 (Prokaryotic annotation) was used to perform the gene prediction [23]. The prediction is relied on the existing annotation resources such proteins and coding DNA sequences (CDS).

### Analysis of single nucleotide polymorphism (SNP)

To identify genetic differences at genome level, the sequencing reads of each strain were mapped to its corresponding reference genome *P*. *aeruginosa* POA1 using Bowtie v0.12.0 software [24]. High-confidence SNP variants data sets were created by applying series of filters. The Variants were identified and extracted using Samtools with the following parameters: each variant must be supported by at least 10 reads (-d = 10), only the variants that were supported by maximum of 10,000 reads were considered for downstream analysis (-D = 10,000) and the reads must be supported by mapping quality of equal or larger than 25 (MQ = 25). Variants harbored by more than 90% of reads were included for further analysis and the depth of coverage was 25x. The effects of nsSNPs were annotated using SnpEFF.

### Genome comparison

Protein Basic Local Alignment Search Tool (BLASTp) was used to match the sequence similarities between genomes of clinical isolates and the *P*. *aeruginosa* reference genome PAO1 [25]. Genome visualization of ten clinical isolates and the similarity between *P*. *aeruginosa* reference genome (NC_002516.2) was performed using BLAST Ring Image Generator (BRIG) [26].

### Heat map

Heat maps were generated using Complex Heat map package from Bioconductor in R [27]. Clusters are predicted using Euclidean distance method.

## Results

### Antibiotic-resistance profiles of *P*. *aeruginosa* clinical isolates

A total of ten *P*. *aeruginosa* clinical isolates with resistant to three or more of the nine antibiotics tested were chosen for the study. The MIC values of nine antibiotics tested against the bacterial isolates are shown in Table 2. Isolates PAS2 and PAS10 were highly resistant to all the antibiotics tested. In contrary, isolates PAS4 and PAS8 were susceptible to at least 3 antibiotics (Imipenem, Meropenem and Collistin). PAS1 and PAS7 were only susceptible to Amikacin and Piperacillin/Tazobactam respectively. All ten isolates were resistant to Ceftazidime, Aztreonam, Gentamicin and Ciprofloxacin.

**Table 2:** Minimum inhibitory concentration (MIC) of the ten *P*. *aeruginosa* clinical isolates used in the current study.

Isolate	IMP (≥32)	MPM (≥32)	CAZ (≥48)	AZT (≥16)	PPT (≥24)	GN (≥32)	AK (≥24)	CIP (≥4)	CL (≥3)
PAS1	**32**	**32**	**256**	**32**	**24**	32	16	**32**	**4**
PAS2	**32**	**32**	**256**	**32**	**32**	**48**	**24**	**32**	**6**
PAS3	2	**32**	**256**	**256**	**256**	**384**	**24**	**32**	2
PAS4	2	3	**256**	**256**	**256**	**1024**	**48**	**32**	2
PAS5	1.5	**32**	**256**	**24**	**128**	**1024**	**192**	**4**	2
PAS6	1.5	3	**256**	**64**	**256**	**1024**	**32**	**32**	**3**
PAS7	**32**	**32**	**256**	**24**	16	**48**	**32**	**32**	**3**
PAS8	1.5	1	**256**	**16**	**256**	**1024**	**98**	**12**	1.5
PAS9	3	1.5	**256**	**16**	**128**	**1024**	**128**	**16**	**6**
PAS10	**32**	**32**	**48**	**16**	**256**	**1024**	**256**	**32**	**4**

Abbreviations: IMP, imipenem; MPM, meropenem; CAZ, ceftazidime; AZT, aztreonam; PPT, piperacillin/tazobactam; GN, gentamicin; AK, amikacin; CIP, ciprofloxacin; CL, colistin.

The bracketed values indicate the breakpoints for the classification of resistance for the respective antibiotics.

### Genome sequencing and assembly

The sequencing consisted of 1 lane 100 bp paired-end reads, yielded approximately 0.8 Gbp to 2.8 Gbp for each clinical isolates. The draft genome assemblies for the ten Pseudomonas clinical isolates have been submitted to NCBI bioproject under the project accession number PRJNA388357 (http://www.ncbi.nlm.nih.gov/bioproject/388357). More than 80% of the reads are above Phred quality score of 30 indicating high quality sequencing data. The sequenced data were subjected to contamination screening and the genome sizes of final assemblies were ranging from 6.6 Mbp to 7.0 Mbp. The de novo assembly of genome sequence data revealed that the number of contigs (>200 bp) varied from 185 to 492 for each genome. These contigs were aligned to the reference genome of *P*. *aeruginosa* PAO1, and manual genome finishing was performed. The maximum contig size among the genomes was 485,560 bp aligned to PAS4. Genomes with the mean identity ranged from 98.77 for PAS 7 to 98.83 for PAS 9. The GC content ranged from 65.79 for PAS 1 to 66.19 for PAS 2. A summary of the genome sequence data and assembly are shown in Table 3.

**Table 3:** Illumina sequencing results for *P*. *aeruginosa* isolates.

Strain	Number of contig	Genome Size (bp)	Reads per pair	Minimum Contig Size (bp)	Maximum Contig Size (bp)	N50 Size (bp)	% of≥ Q30 bases	GC Content (%)	Completeness (%)	Average Identity (%)
**PAS1**	325	6,909,968	28282644	200	404,308	102,152	84.02	65.79	80.34	98.82
**PAS2**	243	6,627,283	25135336	200	379,250	119,180	84.25	66.19	79.06	98.79
**PAS3**	322	6,731,789	20991654	200	327,510	102,161	91.29	66.12	79.68	98.80
**PAS4**	185	6,753,415	8898394	205	485,560	118,034	85.17	66.03	79.80	98.80
**PAS5**	230	6,954,778	9978228	200	404,098	105,908	84.81	65.93	81.22	98.81
**PAS6**	189	7,036,089	7154400	200	377,351	113,718	84.42	65.99	81.21	98.82
**PAS7**	492	6,851,779	10743970	200	319,361	91,918	84.64	65.90	80.25	98.77
**PAS8**	293	6,762,669	8407562	205	238,159	90,287	84.73	66.00	79.61	98.81
**PAS9**	279	7,050,477	8326856	200	363,660	102,446	83.79	65.85	81.65	98.83
**PAS10**	239	6,956,100	27086434	200	377,138	93,007	83.63	65.93	81.21	98.82

### Genome architecture

**Fig 1:**
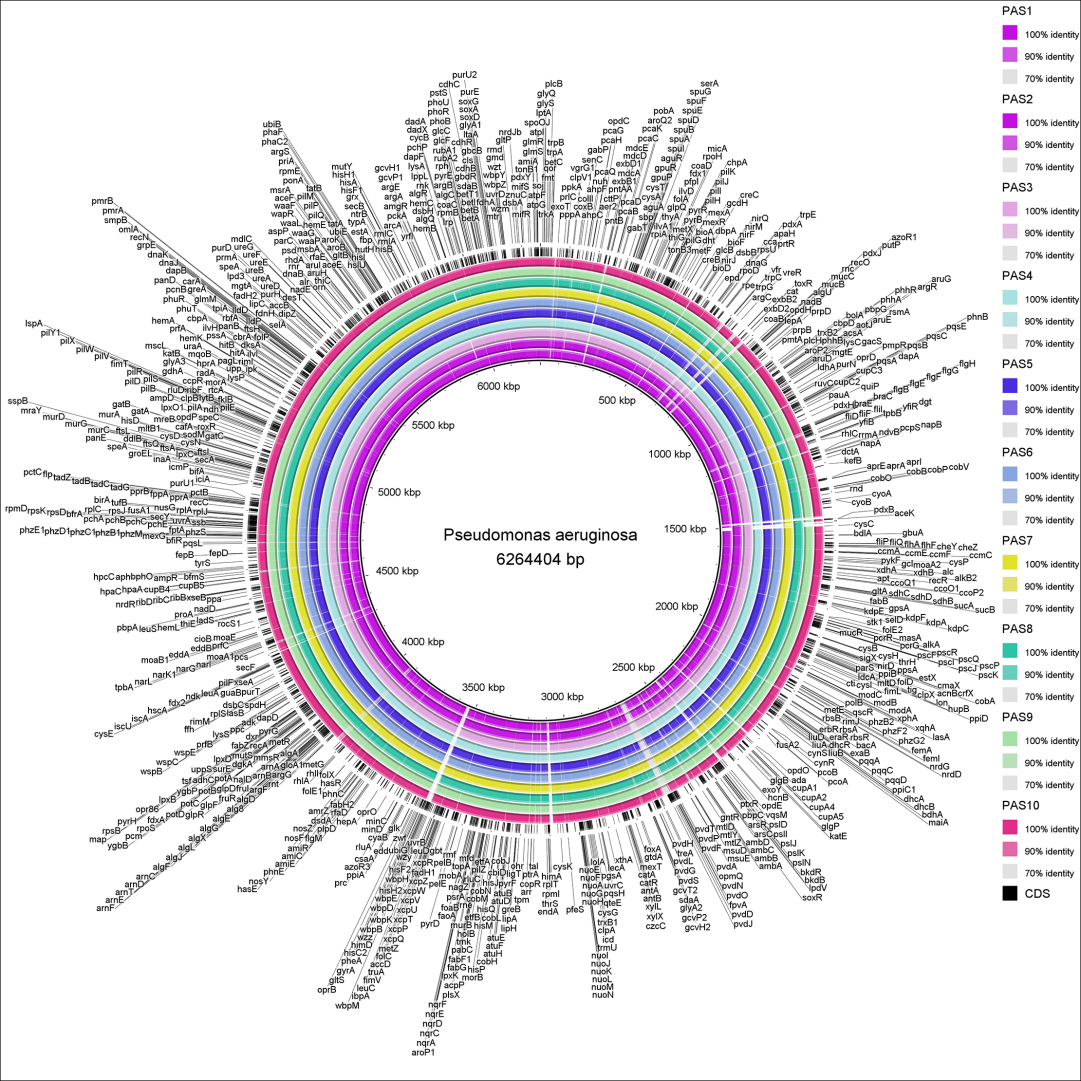
Genome map of clinical isolates compared to *P*. *aeruginosa* reference genome (NC_002516.2). All ten isolates were separated in rings. The inner most circle represents the reference genome and outer most circle with labels represent the CDS (in dark). The percentage similarity between each genome are represented in different colors. Map and underlying analysis were performed with the BLAST Ring Image Generator (BRIG) (http://sourceforge.net/projects/brig).

Fig 1 contains the circular map of the ten newly sequenced clinical isolates compared to the reference genome PAO1. The inner most circle represents the reference genome of *P*. *aeruginosa* (NC_002516.2) and outer most circle with labels represent the protein-coding regions (CDS) with annotation on the genome. The shared identity of each isolate with the reference genome is represented in different colors, which denotes the BLASTn matches between 70% to 100% nucleotide identities. The blank spaces in the rings represent the matches less than 70% or no BLAST matches to the reference genome. The nsSNPs harbored on the genes compared to the reference genome are represented as individual gene names.

### Genome polymorphism

**Fig 2:**
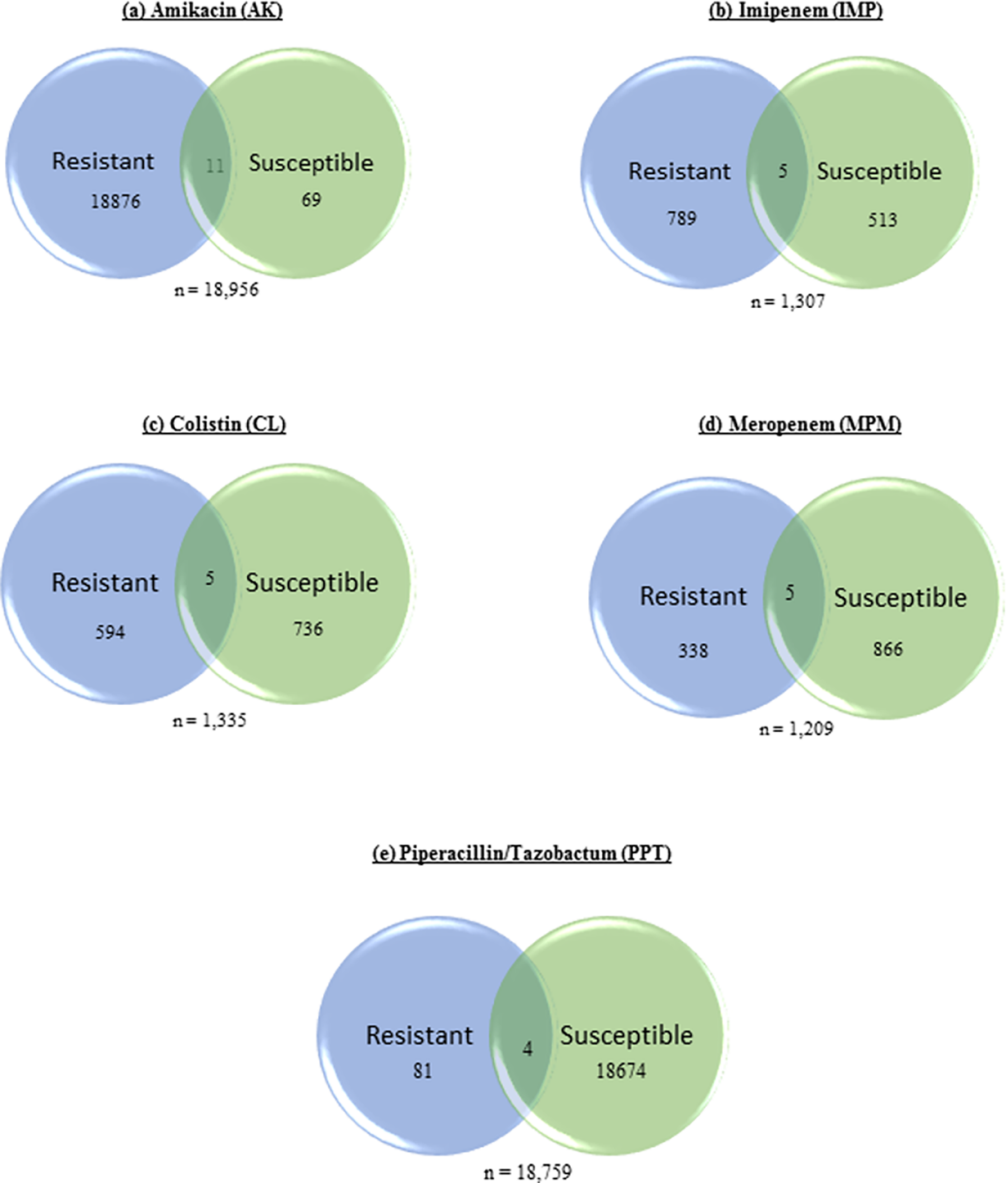
Venn diagram showing the non-synonymous single nucleotide polymorphism (nsSNP’s) in antibiotic resistant and susceptible clinical isolates aligned to the reference genome PAO1. The unique nsSNP’s identified in (a) Amikacin, (b) Imipenem, (c) Colistin, (d) Meropenem and (e) Peperacillin/Tazobactam resistant and susceptible isolates were depicted in the figure. The profiles of antibiotics Ceftazidime, Aztreonam, Gentamicin and Ciprofloxacin were not included in the comparison since all ten clinical isolates were resistant to these antibiotics.

We focused our analysis on identifying nsSNPs that results in amino acid changes within the functional gene sequences. In addition, we also screened for point mutations that leads to premature termination of codons or nucleotide deletions that create frame-shift mutations. We initially screened the nsSNPs in clinical isolates compared to the *P*. *aeruginosa* reference genome, PAO1. Fig 1 shows the genome organization of all the clinical isolates. The CDS regions (in dark) and the genes harboring these regions containing nsSNPs were illustrated in Fig 1. As high as 18,876 nsSNPs were identified in Amikacin resistant isolates when compared to the PAO1 genome; whereas, only 81 nsSNPs were identified in Piperacillin resistant isolates (Fig 2). Majority of the nsSNPs were present within the PA2018, PA2019 and PA5471 loci that code for aminoglycoside resistant genes *mexAB-oprM* and *mexXY-oprM* responsible for the regulation of multi-drug efflux pump (Table 4).

**Table 4:** Key nsSNPs identified in the antibiotic resistant genes of *P*. *aeruginosa* multi-drug resistant isolates compared to PA01 reference genome.

SNP in isolate	SNP in PAO1	Position in PAO1	PAO1 locus	PAO1 amino acid	SNP amino acid	Amino acid position	Protein
G	T	2208200	PA2018	N	T	1036	Resistance–Nodulation-Cell division (RND) multidrug efflux transporter
C	G	2208789	PA2018	Q	E	840	Resistance–Nodulation-Cell division (RND) multidrug efflux transporter
T	C	2209400	PA2018	S	N	636	Resistance–Nodulation-Cell division (RND) multidrug efflux transporter
G	C	2209541	PA2018	G	A	589	Resistance–Nodulation-Cell division (RND) multidrug efflux transporter
C	T	2209680	PA2018	T	A	543	Resistance–Nodulation-Cell division (RND) multidrug efflux transporter
C	T	2209701	PA2018	I	V	536	Resistance–Nodulation-Cell division (RND) multidrug efflux transporter
G	A	2211441	PA2019	W	R	358	Resistance–Nodulation-Cell division (RND) multidrug efflux membrane fusion protein
C	A	2211522	PA2019	L	V	331	Resistance–Nodulation-Cell division (RND) multidrug efflux membrane fusion protein
G	T	2211528	PA2019	K	Q	329	Resistance–Nodulation-Cell division (RND) multidrug efflux membrane fusion protein
C	T	6159991	PA5471	I	V	237	ArmZ
T	C	6160365	PA5471	S	N	112	ArmZ
G	A	6160582	PA5471	C	R	40	ArmZ
A	G	4722060	PA4218	L	F	267	AmpP
C	T	34646	PA0032	L	P	8	Probable Transcriptional regulator
T	C	1286548	PA1184	A	T	81	Probable Transcriptional regulator
T	G	5959101	PA5293	D	E	75	Probable Transcriptional regulator
A	C	4135835	PA3693	A	S	47	Macro domain-containing protein
G	A	2635770	PA2383	S	P	68	Probable Transcriptional regulator
G	A	6159972	PA5471	V	A	243	ArmZ
G	A	2211441	PA2019	W	R	358	Resistance–Nodulation-Cell division (RND) multidrug efflux membrane fusion protein
A	G	2213177	PA2020	W	*	167	MexZ
C	T	5959144	PA5293	Q	R	61	Probable Transcriptional regulator

* Indicates mutation that leads to a stop codon

Then we compared the resistant clinical isolates with the susceptible strains to extract specific nsSNPs found only in resistant isolates. The antibiotic profiles of Ceftazidime, Aztreonam, Gentamicin and Ciprofloxacin were not included as all 10 isolates were resistant to these antibiotics. The isolates classified as resistant for 5 different antibiotics were screened further to identify the nsSNPs. For example, PAS 1 was susceptible to Amikacin and 9 isolates (PAS 2–10) were resistant to the antibiotic. Hence we compared the nsSNPs present within resistant isolates. As shown in S1 Table, the amikacin resistant isolates possess two unique single nucleotide deletions that results in a frameshift mutation that truncates the protein products. Deletion at nucleotide position 1275766 results in a frameshift mutation and truncation of protein NapA (periplasmic nitrate reductase protein). Similarly, deletion at nucleotide position 1402975 results in truncation of 3-mercaptopyruvate sulfurtransferase. The detailed list of unique nsSNPs identified in the resistant clinical isolates compared to susceptible isolates for 5 different antibiotics is shown in the S1 Table. S2 Table, S3 Table, S4 Table and S5 Table. Differential mutation screening between the isolates were performed to identify specific nsSNPs associated with the particular antibiotic resistances.

**Fig 3:**
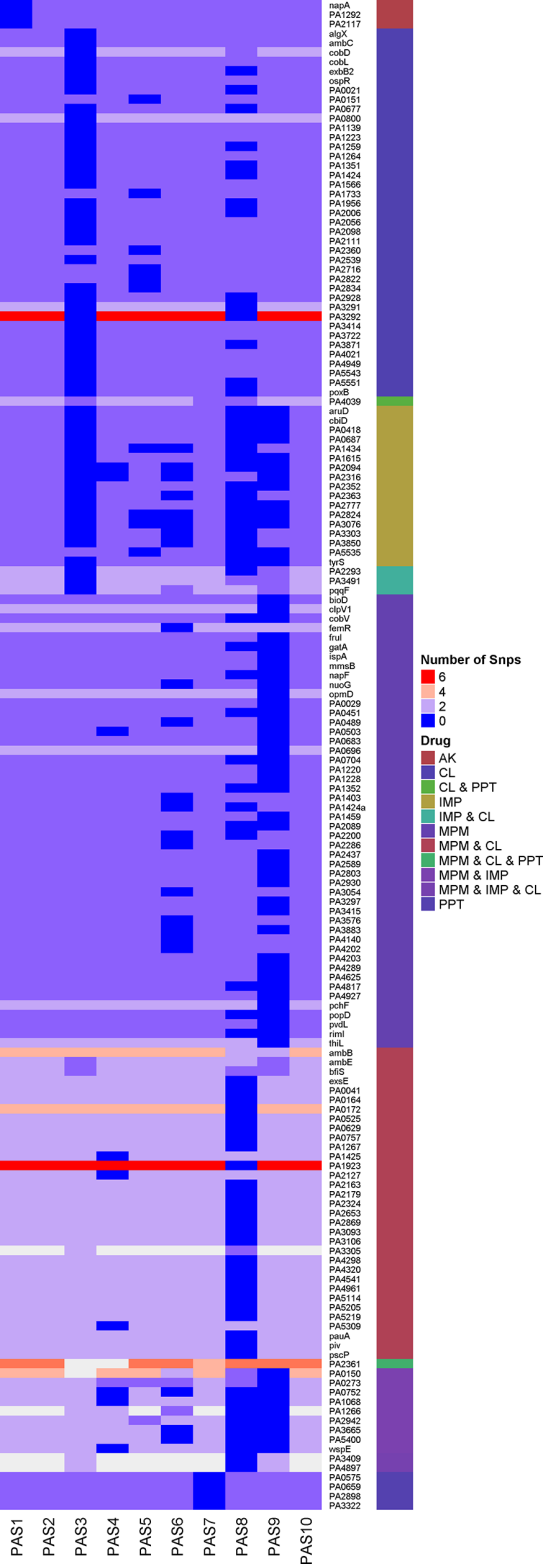
Heat-map representing the number of non-synonymous SNPs found within different antibiotic-resistant clinical isolates of *P*. *aeruginosa*. The number of SNP’s identified in each clinical isolate with respect to their antibiotic resistant profile is depicted in the heat-map. The red colour indicates there are 6 nsSNPs harbouring in that gene among different isolates and a blue colour represents there are no nsSNPs within the gene among isolates.

In order to investigate nsSNPs in genes annotated to be associated in antibiotic resistance [8] or virulence factors [28], we specifically extracted the nsSNPs presented in various antibiotic resistant genes of resistant and susceptible clinical isolates. The number of nsSNPs shared within a gene across all clinical isolates is shown in Fig 3. For example, 6 nsSNPs were found within the colistin resistant isolate (PAS1 and 2) when compared to colistin susceptible isolates (PAS 3 and 8) at the gene PA3292 that codes for a hypothetical protein. Similarly, 6 nsSNPs were also seen in all the isolates except PAS 8 at the PA1923 gene that codes for cobaltochelatase subunit CobN. Many of the polymorphic genes among these clinical isolates were mainly associated with cell wall, multi-drug efflux pump, protein secretion, DNA replication and genes involved in repair mechanism. In addition, nsSNPs were found within genes responsible for resistance to β–lactams; transporter (PA4218), transcriptional regulators (PA1184, PA0032, PA5293 and PA2383) and a hypothetical protein (PA3693). These were all collectively reported to play a role in decreased antibiotic uptake and affecting cell wall permeability [29]. Notably, a SNP at position 2213177 in all antibiotic resistant isolates changed tryptophan into a stop codon. Furthermore, two frame shift mutations at the *napA* and *PA1292* genes of Amikacin resistant genes and a mutation resulting in stop codon at amino acid position Q65 in the Meropenem resistant isolates might have implications in the drug resistant mechanism. These findings indicate that the nsSNPs identified in the clinical isolates may contribute towards antibiotic resistance between the resistant clinical isolates examined in the study. Further targeted evidence using a large cohort of clinical isolates and site directed mutational analysis of these antibiotic resistant genes will provide further insights in the antibiotic resistant mechanism of the *P*. *aeruginosa*.

## Discussion

Whole genome sequencing promises high resolution, high-throughput genome sequencing with insights to the repertoire of genetic polymorphism[3, 11]. This approach can be used to identify the single nucleotide polymorphism that either alters a single amino acid or leads to a stop codon, and frame-shift mutations that alters a gene sequence itself resulting in truncated proteins. Here, we have investigated the genetic variation in *P*. *aeruginosa* hospital isolates by high-throughput NGS technology and comparative genomics.

The clinical isolates included in this study were selected based on their multidrug resistant profiles against a total of nine antibiotics. To study the genomic variation among the isolates with varying antibiotic resistance characteristics, we analyzed the genomic sequences of the isolates using WGS technique and compared the data with *P*. *aeruginosa* reference genome PAO1.

Several non-synonymous SNPs that may play an important role in antibiotic resistance have been identified, with insights to enhance our current understanding of the factors that influence the antibiotic resistance regime of hospital isolates. Though we have studied the nsSNPs of entire genome of all hospital isolates, we specifically extracted nsSNPs that were in genes reported to play a major implication in antibiotic resistance [28] within the antibiotic resistant and susceptible clinical isolates. Several mechanisms of antibiotic resistance in *P*. *aeruginosa* have been reported: by altering porins or outer membrane properties, resistance through enzymatic modification of drugs, and resistance through efflux systems and chromosomal mutations within regulatory genes [30–33].

We have identified specific nsSNPs within the *mexXY-oprM* and *mexAB-oprM* genes, which are known to play a major role in antibiotic resistance through efflux pumps and both these genes part the same efflux porin OprM [34, 35]. In addition, the efflux system MexXY-OprM is also recognized to be contributing in aminoglycoside resistance in *P*. *aeruginosa*, and MexAB-OprM is reported to confer resistance to β—lactams, aminoglycosides and fluoroquinolones resistance [31, 36]. Also a polymorphism resulting in a stop codon at locus PA2020 corresponding to a *mexZ* transcriptional regulator may have implications in drug resistance [37]. Collectively, *P*. *aeruginosa* may use a combination of all these mechanisms to achieve multi-drug antibiotic resistance.

The unique nsSNPs identified within the genes coding for aminoglycoside modifying enzymes (AME’s) and 16S rRNA methylase genes, mutations leading to colistin resistance by altering the bacterial outer membrane, and resistant to β lactam by hyper production of β-lactamase AmpC may together play a crucial role in multi-drug resistance. Our results confirm the assumption of other authors that the drug resistance may be the result of a pool of pathogenicity-related genes that interact in various combinations [29, 38, 39]. In addition to the unique mutations, there is high degree of sequence conservation between resistant and susceptible clinical isolates. This conservation within the virulence genes of different *P*. *aeruginosa* clinical isolates may be a factor that is challenging to device strategies for the treatment of infections. In addition, the extensive conservation of virulence genes in the genomes regardless of drug resistance may be due to the fact that the disease-causing ability of this opportunistic pathogen relies on a set of highly conserved pathogenic mechanisms [29]. Moreover, the resistance of one isolate for several antibiotics may be due to the pleiotropic effects of resistance causing gene [39].

Drug resistance to several antibiotics may be due to a combination of mutations resulting in overexpression of several multi-drug efflux pumps, alteration of expression of enzymes and structural components involved in peptidoglycan outer membrane stability, mutations affecting gyrase activity and mutations taking effect in aminoglycoside phosphotransferases [29]. In our study, the analysis of the genomes of drug resistant clinical isolates possessed non-synonymous mutations in at least one gene known to be involved in antibiotic resistance (for example, oprD, gyrA or B, mex-type efflux systems, parC, rpoB, baeS). The genes carrying these mutations are coding for proteins involved in outer membrane permeability, multi- drug efflux pumps, gyrases and drug modifying enzymes (aminoglycoside modification enzymes and/ or β -lactamases) [39]. However, a limitation of this study is that further targeted evidence with site directed mutational analysis on the reported nsSNPs harboring the resistant genes is required. Such analysis will provide further insights to the overall phenotype alteration and synergistic effect of these nsSNPs in the antibiotic resistant mechanism.

In conclusion, this study demonstrates the potential of next generation high-throughput whole genome sequencing to compare the genome polymorphism between clinical isolates. We have identified a number of key mutations that may play a key role in altering antibiotic resistant genes leading to genetic polymorphism. This should form the basis of our future research to study the prevalence of such multidrug resistance *P*. *aeruginosa* in the Asia pacific region. This technology can be utilised in conjunction with current epidemiological studies, diagnostic assays and antimicrobial susceptibility tests to reveal the genetic variation and pathogen biology of “high-risk” pathogens including *P*. *aeruginosa* [11].

## Supporting information

S1 Table Non-synonymous SNP’s identified in Amikacin resistant isolates. The Amikacin susceptible clinical isolate PAS1 was compared against all other resistant isolates.
(DOCX)

S2 Table Non-synonymous SNP’s in Meropenem resistant isolates. The Meropenem susceptible clinical isolates PAS 4, 6, 8 and 9 were compared against the rest of the resistant isolates.
(DOCX)

S3 Table Non-synonymous SNP’s in Imipenem resistant isolates. The Imipenem resistant isolates PAS 1, 2, 7 and 10 were compared against the rest of the susceptible isolates.
(DOCX)

S4 Table Non-synonymous SNP’s in Colistin resistant isolates. The Colistin susceptible isolates PAS 3, 4, 5 and 8 were compared against the rest of the resistant isolates.
(DOCX)

S5 Table Non-synonymous SNP’s in Piperacillin/Tazobactam resistant isolates. The Tazobactam susceptible isolate PAS 7 was compared against the rest of the resistant isolates.
(DOCX)
