# Supplementary material for: Next generation sequencing reveals the antibiotic resistant variants in the genome of Pseudomonas aeruginosa
Source: PLoS One. 2017 Aug 10;12(8):e0182524. doi: 10.1371/journal.pone.0182524 (PMC5557631; doi:10.1371/journal.pone.0182524)
Supplement: S1 Table — The Amikacin susceptible clinical isolate PAS1 was compared against all other resistant isolates. (DOCX) [file pone.0182524.s001.docx]

**S1 Table. Non-synonymous SNP’s identified in Amikacin resistant isolates.** Amikacin susceptible clinical isolate PAS1 was compared against all other resistant isolates.

| S. No | **Nucleotide Position** | **Susceptible genome** | **Alteration** | **Gene ID** | **AA changes** | **Hydrophobicity** | **Charges** | **Polarity** | **Sequence length in reference** | **Sequence length in isolate** | **Nucleotide difference** |
| --- | --- | --- | --- | --- | --- | --- | --- | --- | --- | --- | --- |
| 1 | 1275766 | GAA | GA | napA | * | - | - | - | - | - | -11 |
| 2 | 1402975 | GCGTCGTC | GCGTC | PA1292 | * | - | - | - | - | - | -285 |
| 3 | 2328283 | T | C | PA2117 | I292V | hydrophobic-hydrophobic | neutral-neutral | Non-polar-Non-polar | 131 | 117 | 14 |

* - Indicates a frame shift mutation
