# Supplementary material for: Next generation sequencing reveals the antibiotic resistant variants in the genome of Pseudomonas aeruginosa
Source: PLoS One. 2017 Aug 10;12(8):e0182524. doi: 10.1371/journal.pone.0182524 (PMC5557631; doi:10.1371/journal.pone.0182524)
Supplement: S2 Table — The Meropenem susceptible clinical isolates PAS 4, 6, 8 and 9 were compared against the rest of the resistant isolates. (DOCX) [file pone.0182524.s002.docx]

**S2 Table. Non-synonymous SNP’s in Meropenem resistant isolates.** The Meropenem susceptible clinical isolates PAS 4, 6, 8 and 9 were compared against the rest of the resistant isolates.

| S. No | **Nucleotide Position** | **Susceptible genome** | **Alteration** | **Gene ID** | **AA changes** | **Hydrophobicity** | **Charges** | **Polarity** | **Sequence length in reference** | **Sequence length in isolate** | **Nucleotide difference** |
| --- | --- | --- | --- | --- | --- | --- | --- | --- | --- | --- | --- |
| 1 | 32000 | A | G | PA0029 | M19T | hydrophobic-hydrophilic | neutral-neutral | Non-polar-Polar | 149 | 119 | 30 |
| 2 | 49850 | A | G | PA0041 | T2313A | hydrophilic-hydrophobic | neutral-neutral | Polar-Non-polar | 119 | 89 | 30 |
| 3 | 109791 | T | C | clpV1 | V524A | hydrophobic-hydrophobic | neutral-neutral | Non-polar-Non-polar | 117 | 89 | 28 |
| 4 | 109812 | T | C | clpV1 | V531A | hydrophobic-hydrophobic | neutral-neutral | Non-polar-Non-polar | 117 | 89 | 28 |
| 5 | 170434 | G | A | PA0150 | A178T | hydrophobic-hydrophilic | neutral-neutral | Non-polar-Polar | 89 | 119 | 30 |
| 6 | 170516 | C | A | PA0150 | A205E | hydrophobic-hydrophilic | neutral-negative | Non-polar-Polar | 89 | 147 | 58 |
| 7 | 187205 | G | C | PA0164 | G125A | hydrophobic-hydrophobic | neutral-neutral | Non-polar-Non-polar | 75 | 89 | 14 |
| 8 | 196376 | G | T | PA0172 | R125S | hydrophilic-hydrophilic | positive-neutral | Polar-Polar | 174 | 105 | 69 |
| 9 | 196398 | G | C | PA0172 | H117Q | hydrophilic-hydrophilic | positive-neutral | Polar-Polar | 155 | 146 | 9 |
| 10 | 308512 | T | G | PA0273 | D194A | hydrophilic-hydrophobic | negative-neutral | Polar-Non-polar | 133 | 89 | 44 |
| 11 | 508255 | A | G | PA0451 | S209G | hydrophilic-hydrophobic | neutral-neutral | Polar-Non-polar | 105 | 75 | 30 |
| 12 | 550312 | G | A | PA0489 | R24C | hydrophilic-hydrophobic | positive-neutral | Polar-Non-polar | 174 | 121 | 53 |
| 13 | 562928 | T | C | PA0503 | Y70H | hydrophilic-hydrophilic | neutral-positive | Polar-Polar | 181 | 155 | 26 |
| 14 | 563718 | T | C | bioD | V57A | hydrophobic-hydrophobic | neutral-neutral | Non-polar-Non-polar | 117 | 89 | 28 |
| 15 | 584592 | A | G | PA0525 | N246D | hydrophilic-hydrophilic | neutral-negative | Polar-Polar | 132 | 133 | 1 |
| 16 | 688201 | G | A | PA0629 | G75S | hydrophobic-hydrophilic | neutral-neutral | Non-polar-Polar | 75 | 105 | 30 |
| 17 | 740468 | A | G | PA0683 | E89G | hydrophilic-hydrophobic | negative-neutral | Polar-Non-polar | 147 | 75 | 72 |
| 18 | 768607 | C | T | PA0696 | Q65** | hydrophilic- | neutral- | Polar- | 146 |  | 146 |
| 19 | 768608 | A | T | PA0696 | Q65L | hydrophilic-hydrophobic | neutral-neutral | Polar-Non-polar | 146 | 131 | 15 |
| 20 | 777692 | T | C | PA0704 | T164A | hydrophilic-hydrophobic | neutral-neutral | Polar-Non-polar | 119 | 89 | 30 |
| 21 | 820826 | T | A | PA0752 | S183C | hydrophilic-hydrophobic | neutral-neutral | Polar-Non-polar | 105 | 121 | 16 |
| 22 | 825396 | T | G | PA0757 | D108E | hydrophilic-hydrophilic | negative-negative | Polar-Polar | 133 | 147 | 14 |
| 23 | 1102874 | T | C | pauA | F649L | hydrophobic-hydrophobic | neutral-neutral | Non-polar-Non-polar | 165 | 131 | 34 |
| 24 | 1154339 | T | C | PA1068 | S235P | hydrophilic-hydrophobic | neutral-neutral | Polar-Non-polar | 105 | 115 | 10 |
| 25 | 1276523 | G | T | napF | A29E | hydrophobic-hydrophilic | neutral-negative | Non-polar-Polar | 89 | 147 | 58 |
| 26 | 1321163 | T | C | PA1220 | H365R | hydrophilic-hydrophilic | positive-positive | Polar-Polar | 155 | 174 | 19 |
| 27 | 1330238 | A | G | PA1228 | L77P | hydrophobic-hydrophobic | neutral-neutral | Non-polar-Non-polar | 131 | 115 | 16 |
| 28 | 1375900 | C | G | PA1266 | Q66H | hydrophilic-hydrophilic | neutral-positive | Polar-Polar | 146 | 155 | 9 |
| 29 | 1377249 | G | C | PA1267 | A65G | hydrophobic-hydrophobic | neutral-neutral | Non-polar-Non-polar | 89 | 75 | 14 |
| 30 | 1393736 | C | G | cobV | V112L | hydrophobic-hydrophobic | neutral-neutral | Non-polar-Non-polar | 117 | 131 | 14 |
| 31 | 1466701 | T | C | PA1352 | D207G | hydrophilic-hydrophobic | negative-neutral | Polar-Non-polar | 133 | 75 | 58 |
| 32 | 1525943 | A | G | PA1403 | T93A | hydrophilic-hydrophobic | neutral-neutral | Polar-Non-polar | 119 | 89 | 30 |
| 33 | 1550777 | G | A | PA1424a | P4L | hydrophobic-hydrophobic | neutral-neutral | Non-polar-Non-polar | 115 | 131 | 16 |
| 34 | 1551303 | C | A | PA1425 | A107D | hydrophobic-hydrophilic | neutral-negative | Non-polar-Polar | 89 | 133 | 44 |
| 35 | 1590053 | A | G | PA1459 | N239S | hydrophilic-hydrophilic | neutral-neutral | Polar-Polar | 132 | 105 | 27 |
| 36 | 1844667 | A | G | pscP | V196A | hydrophobic-hydrophobic | neutral-neutral | Non-polar-Non-polar | 117 | 89 | 28 |
| 37 | 1855018 | T | C | popD | V57A | hydrophobic-hydrophobic | neutral-neutral | Non-polar-Non-polar | 117 | 89 | 28 |
| 38 | 1856415 | G | C | exsE | E36D | hydrophilic-hydrophilic | negative-negative | Polar-Polar | 147 | 133 | 14 |
| 39 | 2085413 | T | C | femR | D5G | hydrophilic-hydrophobic | negative-neutral | Polar-Non-polar | 133 | 75 | 58 |
| 40 | 2085420 | C | A | femR | A3S | hydrophobic-hydrophilic | neutral-neutral | Non-polar-Polar | 89 | 105 | 16 |
| 41 | 2099646 | G | C | PA1923 | E65D | hydrophilic-hydrophilic | negative-negative | Polar-Polar | 147 | 133 | 14 |
| 42 | 2099662 | A | G | PA1923 | N71D | hydrophilic-hydrophilic | neutral-negative | Polar-Polar | 132 | 133 | 1 |
| 43 | 2099713 | G | C | PA1923 | E88Q | hydrophilic-hydrophilic | negative-neutral | Polar-Polar | 147 | 146 | 1 |
| 44 | 2298301 | A | G | PA2089 | Q97R | hydrophilic-hydrophilic | neutral-positive | Polar-Polar | 146 | 174 | 28 |
| 45 | 2341243 | G | C | PA2127 | A133G | hydrophobic-hydrophobic | neutral-neutral | Non-polar-Non-polar | 89 | 75 | 14 |
| 46 | 2384930 | C | T | PA2163 | R558Q | hydrophilic-hydrophilic | positive-neutral | Polar-Polar | 174 | 146 | 28 |
| 47 | 2400663 | A | G | PA2179 | S315P | hydrophilic-hydrophobic | neutral-neutral | Polar-Non-polar | 105 | 115 | 10 |
| 48 | 2419278 | A | C | PA2200 | S186R | hydrophilic-hydrophilic | neutral-positive | Polar-Polar | 105 | 174 | 69 |
| 49 | 2514116 | G | A | PA2286 | S92N | hydrophilic-hydrophilic | neutral-neutral | Polar-Polar | 105 | 132 | 27 |
| 50 | 2535302 | T | G | ambE | T1248P | hydrophilic-hydrophobic | neutral-neutral | Polar-Non-polar | 119 | 115 | 4 |
| 51 | 2541237 | A | G | ambB | I1237T | hydrophobic-hydrophilic | neutral-neutral | Non-polar-Polar | 131 | 119 | 12 |
| 52 | 2542741 | T | C | ambB | S736G | hydrophilic-hydrophobic | neutral-neutral | Polar-Non-polar | 105 | 75 | 30 |
| 53 | 2542764 | C | T | ambB | G728D | hydrophobic-hydrophilic | neutral-negative | Non-polar-Polar | 75 | 133 | 58 |
| 54 | 2565208 | T | C | PA2324 | C307R | hydrophobic-hydrophilic | neutral-positive | Non-polar-Polar | 121 | 174 | 53 |
| 55 | 2611137 | A | G | PA2361 | V303A | hydrophobic-hydrophobic | neutral-neutral | Non-polar-Non-polar | 117 | 89 | 28 |
| 56 | 2717354 | T | C | pvdL | H1114R | hydrophilic-hydrophilic | positive-positive | Polar-Polar | 155 | 174 | 19 |
| 57 | 2733335 | G | C | PA2437 | L276V | hydrophobic-hydrophobic | neutral-neutral | Non-polar-Non-polar | 131 | 117 | 14 |
| 58 | 2930217 | T | C | PA2589 | D194G | hydrophilic-hydrophobic | negative-neutral | Polar-Non-polar | 133 | 75 | 58 |
| 59 | 2989170 | A | G | nuoG | T484A | hydrophilic-hydrophobic | neutral-neutral | Polar-Non-polar | 119 | 89 | 30 |
| 60 | 3002489 | T | C | PA2653 | N237S | hydrophilic-hydrophilic | neutral-neutral | Polar-Polar | 132 | 105 | 27 |
| 61 | 3159304 | A | G | PA2803 | N127D | hydrophilic-hydrophilic | neutral-negative | Polar-Polar | 132 | 133 | 1 |
| 62 | 3221683 | T | C | PA2869 | L11P | hydrophobic-hydrophobic | neutral-neutral | Non-polar-Non-polar | 131 | 115 | 16 |
| 63 | 3286138 | A | G | PA2930 | H151R | hydrophilic-hydrophilic | positive-positive | Polar-Polar | 155 | 174 | 19 |
| 64 | 3298068 | T | C | PA2942 | N285S | hydrophilic-hydrophilic | neutral-neutral | Polar-Polar | 132 | 105 | 27 |
| 65 | 3419693 | G | C | PA3054 | S469T | hydrophilic-hydrophilic | neutral-neutral | Polar-Polar | 105 | 119 | 14 |
| 66 | 3474074 | T | C | PA3093 | S353P | hydrophilic-hydrophobic | neutral-neutral | Polar-Non-polar | 105 | 115 | 10 |
| 67 | 3486431 | T | C | PA3106 | I252V | hydrophobic-hydrophobic | neutral-neutral | Non-polar-Non-polar | 131 | 117 | 14 |
| 68 | 3691551 | T | C | PA3297 | S1152G | hydrophilic-hydrophobic | neutral-neutral | Polar-Non-polar | 105 | 75 | 30 |
| 69 | 3704096 | T | C | PA3305 | T356A | hydrophilic-hydrophobic | neutral-neutral | Polar-Non-polar | 119 | 89 | 30 |
| 70 | 3818184 | A | C | PA3409 | S117A | hydrophilic-hydrophobic | neutral-neutral | Polar-Non-polar | 105 | 89 | 16 |
| 71 | 3822061 | G | C | PA3415 | A146G | hydrophobic-hydrophobic | neutral-neutral | Non-polar-Non-polar | 89 | 75 | 14 |
| 72 | 3992869 | C | T | fruI | A515T | hydrophobic-hydrophilic | neutral-neutral | Non-polar-Polar | 89 | 119 | 30 |
| 73 | 4001645 | C | T | mmsB | V150I | hydrophobic-hydrophobic | neutral-neutral | Non-polar-Non-polar | 117 | 131 | 14 |
| 74 | 4008142 | T | C | PA3576 | S3P | hydrophilic-hydrophobic | neutral-neutral | Polar-Non-polar | 105 | 115 | 10 |
| 75 | 4104365 | A | G | PA3665 | T92A | hydrophilic-hydrophobic | neutral-neutral | Polar-Non-polar | 119 | 89 | 30 |
| 76 | 4147870 | A | T | wspE | L128Q | hydrophobic-hydrophilic | neutral-neutral | Non-polar-Polar | 131 | 146 | 15 |
| 77 | 4350159 | T | C | PA3883 | S220P | hydrophilic-hydrophobic | neutral-neutral | Polar-Non-polar | 105 | 115 | 10 |
| 78 | 4526157 | T | G | ispA | S203A | hydrophilic-hydrophobic | neutral-neutral | Polar-Non-polar | 105 | 89 | 16 |
| 79 | 4533556 | G | C | thiL | D22E | hydrophilic-hydrophilic | negative-negative | Polar-Polar | 133 | 147 | 14 |
| 80 | 4533561 | C | T | thiL | A21T | hydrophobic-hydrophilic | neutral-neutral | Non-polar-Polar | 89 | 119 | 30 |
| 81 | 4630385 | T | C | PA4140 | L4P | hydrophobic-hydrophobic | neutral-neutral | Non-polar-Non-polar | 131 | 115 | 16 |
| 82 | 4671622 | A | G | piv | T102A | hydrophilic-hydrophobic | neutral-neutral | Polar-Non-polar | 119 | 89 | 30 |
| 83 | 4695489 | G | A | bfiS | L183F | hydrophobic-hydrophobic | neutral-neutral | Non-polar-Non-polar | 131 | 165 | 34 |
| 84 | 4702790 | A | G | PA4202 | V103A | hydrophobic-hydrophobic | neutral-neutral | Non-polar-Non-polar | 117 | 89 | 28 |
| 85 | 4703587 | A | G | PA4203 | H128R | hydrophilic-hydrophilic | positive-positive | Polar-Polar | 155 | 174 | 19 |
| 86 | 4711425 | G | A | opmD | G269S | hydrophobic-hydrophilic | neutral-neutral | Non-polar-Polar | 75 | 105 | 30 |
| 87 | 4711429 | C | G | opmD | A270G | hydrophobic-hydrophobic | neutral-neutral | Non-polar-Non-polar | 89 | 75 | 14 |
| 88 | 4733279 | T | C | pchF | T1174A | hydrophilic-hydrophobic | neutral-neutral | Polar-Non-polar | 119 | 89 | 30 |
| 89 | 4733727 | T | G | pchF | L1024F | hydrophobic-hydrophobic | neutral-neutral | Non-polar-Non-polar | 131 | 165 | 34 |
| 90 | 4812590 | A | G | PA4289 | T399A | hydrophilic-hydrophobic | neutral-neutral | Polar-Non-polar | 119 | 89 | 30 |
| 91 | 4823319 | T | G | PA4298 | T16P | hydrophilic-hydrophobic | neutral-neutral | Polar-Non-polar | 119 | 115 | 4 |
| 92 | 4849311 | A | G | PA4320 | T280A | hydrophilic-hydrophobic | neutral-neutral | Polar-Non-polar | 119 | 89 | 30 |
| 93 | 5014393 | G | C | gatA | K140N | hydrophilic-hydrophilic | positive-neutral | Polar-Polar | 146 | 132 | 14 |
| 94 | 5085360 | G | C | PA4541 | S973T | hydrophilic-hydrophilic | neutral-neutral | Polar-Polar | 105 | 119 | 14 |
| 95 | 5191879 | T | C | PA4625 | T333A | hydrophilic-hydrophobic | neutral-neutral | Polar-Non-polar | 119 | 89 | 30 |
| 96 | 5248574 | A | G | rimI | S67P | hydrophilic-hydrophobic | neutral-neutral | Polar-Non-polar | 105 | 115 | 10 |
| 97 | 5407391 | T | A | PA4817 | S6T | hydrophilic-hydrophilic | neutral-neutral | Polar-Polar | 105 | 119 | 14 |
| 98 | 5491479 | C | T | PA4897 | A45V | hydrophobic-hydrophobic | neutral-neutral | Non-polar-Non-polar | 89 | 117 | 28 |
| 99 | 5527119 | T | C | PA4927 | K427E | hydrophilic-hydrophilic | positive-negative | Polar-Polar | 146 | 147 | 1 |
| 100 | 5570022 | A | C | PA4961 | E311D | hydrophilic-hydrophilic | negative-negative | Polar-Polar | 147 | 133 | 14 |
| 101 | 5761034 | G | A | PA5114 | P104S | hydrophobic-hydrophilic | neutral-neutral | Non-polar-Polar | 115 | 105 | 10 |
| 102 | 5859820 | A | G | PA5205 | I10V | hydrophobic-hydrophobic | neutral-neutral | Non-polar-Non-polar | 131 | 117 | 14 |
| 103 | 5877231 | G | T | PA5219 | A190S | hydrophobic-hydrophilic | neutral-neutral | Non-polar-Polar | 89 | 105 | 16 |
| 104 | 5978437 | A | C | PA5309 | T217P | hydrophilic-hydrophobic | neutral-neutral | Polar-Non-polar | 119 | 115 | 4 |
| 105 | 6080878 | T | C | PA5400 | V252A | hydrophobic-hydrophobic | neutral-neutral | Non-polar-Non-polar | 117 | 89 | 28 |

** - Indicates a mutation that resulted in a stop codon.
