# Supplementary material for: Next generation sequencing reveals the antibiotic resistant variants in the genome of Pseudomonas aeruginosa
Source: PLoS One. 2017 Aug 10;12(8):e0182524. doi: 10.1371/journal.pone.0182524 (PMC5557631; doi:10.1371/journal.pone.0182524)
Supplement: S3 Table — The Imipenem resistant isolates PAS 1, 2, 7 and 10 were compared against the rest of the susceptible isolates. (DOCX) [file pone.0182524.s003.docx]

**S3 Table. Non-synonymous SNP’s in Imipenem resistant isolates.** The Imipenem resistant isolates PAS 1, 2, 7 and 10 were compared against the rest of the susceptible isolates.

| S. No | **Nucleotide Position** | **Susceptible genome** | **Alteration** | **Gene ID** | **AA changes** | **Hydrophobicity** | **Charges** | **Polarity** | **Sequence length in reference** | **Sequence length in isolate** | **Nucleotide difference** |
| --- | --- | --- | --- | --- | --- | --- | --- | --- | --- | --- | --- |
| 1 | 170516 | C | A | PA0150 | A205E | hydrophobic-hydrophilic | neutral-negative | Non-polar-Polar | 89 | 147 | 58 |
| 2 | 170627 | A | G | PA0150 | K242R | hydrophilic-hydrophilic | positive-positive | Polar-Polar | 146 | 174 | 28 |
| 3 | 307991 | C | T | PA0273 | A368T | hydrophobic-hydrophilic | neutral-neutral | Non-polar-Polar | 89 | 119 | 30 |
| 4 | 465623 | T | C | PA0418 | N121D | hydrophilic-hydrophilic | neutral-negative | Polar-Polar | 132 | 133 | 1 |
| 5 | 745889 | A | G | PA0687 | T50A | hydrophilic-hydrophobic | neutral-neutral | Polar-Non-polar | 119 | 89 | 30 |
| 6 | 820826 | T | A | PA0752 | S183C | hydrophilic-hydrophobic | neutral-neutral | Polar-Non-polar | 105 | 121 | 16 |
| 7 | 981536 | A | G | aruD | T39A | hydrophilic-hydrophobic | neutral-neutral | Polar-Non-polar | 119 | 89 | 30 |
| 8 | 1154339 | T | C | PA1068 | S235P | hydrophilic-hydrophobic | neutral-neutral | Polar-Non-polar | 105 | 115 | 10 |
| 9 | 1375590 | C | T | PA1266 | V170I | hydrophobic-hydrophobic | neutral-neutral | Non-polar-Non-polar | 117 | 131 | 14 |
| 10 | 1375900 | C | G | PA1266 | Q66H | hydrophilic-hydrophilic | neutral-positive | Polar-Polar | 146 | 155 | 9 |
| 11 | 1562370 | C | T | PA1434 | R88Q | hydrophilic-hydrophilic | positive-neutral | Polar-Polar | 174 | 146 | 28 |
| 12 | 1760043 | C | G | PA1615 | T87S | hydrophilic-hydrophilic | neutral-neutral | Polar-Polar | 119 | 105 | 14 |
| 13 | 2157472 | G | A | pqqF | R700Q | hydrophilic-hydrophilic | positive-neutral | Polar-Polar | 174 | 146 | 28 |
| 14 | 2305298 | A | T | PA2094 | M159L | hydrophobic-hydrophobic | neutral-neutral | Non-polar-Non-polar | 149 | 131 | 18 |
| 15 | 2523949 | C | G | PA2293 | V85L | hydrophobic-hydrophobic | neutral-neutral | Non-polar-Non-polar | 117 | 131 | 14 |
| 16 | 2554466 | G | A | PA2316 | A168T | hydrophobic-hydrophilic | neutral-neutral | Non-polar-Polar | 89 | 119 | 30 |
| 17 | 2598742 | T | C | PA2352 | F15L | hydrophobic-hydrophobic | neutral-neutral | Non-polar-Non-polar | 165 | 131 | 34 |
| 18 | 2613455 | T | C | PA2363 | I232V | hydrophobic-hydrophobic | neutral-neutral | Non-polar-Non-polar | 131 | 117 | 14 |
| 19 | 3135907 | T | C | PA2777 | T32A | hydrophilic-hydrophobic | neutral-neutral | Polar-Non-polar | 119 | 89 | 30 |
| 20 | 3178359 | A | G | PA2824 | I424V | hydrophobic-hydrophobic | neutral-neutral | Non-polar-Non-polar | 131 | 117 | 14 |
| 21 | 3264091 | A | G | cbiD | Q78R | hydrophilic-hydrophilic | neutral-positive | Polar-Polar | 146 | 174 | 28 |
| 22 | 3298000 | T | C | PA2942 | T308A | hydrophilic-hydrophobic | neutral-neutral | Polar-Non-polar | 119 | 89 | 30 |
| 23 | 3449821 | A | C | PA3076 | D42A | hydrophilic-hydrophobic | negative-neutral | Polar-Non-polar | 133 | 89 | 44 |
| 24 | 3701721 | A | G | PA3303 | H262R | hydrophilic-hydrophilic | positive-positive | Polar-Polar | 155 | 174 | 19 |
| 25 | 3818399 | C | G | PA3409 | R45P | hydrophilic-hydrophobic | positive-neutral | Polar-Non-polar | 174 | 115 | 59 |
| 26 | 3910662 | G | C | PA3491 | A751P | hydrophobic-hydrophobic | neutral-neutral | Non-polar-Non-polar | 89 | 115 | 26 |
| 27 | 4104365 | A | G | PA3665 | T92A | hydrophilic-hydrophobic | neutral-neutral | Polar-Non-polar | 119 | 89 | 30 |
| 28 | 4147870 | A | T | wspE | L128Q | hydrophobic-hydrophilic | neutral-neutral | Non-polar-Polar | 131 | 146 | 15 |
| 29 | 4311423 | C | G | PA3850 | R147P | hydrophilic-hydrophobic | positive-neutral | Polar-Non-polar | 174 | 115 | 59 |
| 30 | 4628027 | T | C | tyrS | T387A | hydrophilic-hydrophobic | neutral-neutral | Polar-Non-polar | 119 | 89 | 30 |
| 31 | 5491503 | A | G | PA4897 | D53G | hydrophilic-hydrophobic | negative-neutral | Polar-Non-polar | 133 | 75 | 58 |
| 32 | 6080878 | T | C | PA5400 | V252A | hydrophobic-hydrophobic | neutral-neutral | Non-polar-Non-polar | 117 | 89 | 28 |
| 33 | 6228357 | C | T | PA5535 | A363T | hydrophobic-hydrophilic | neutral-neutral | Non-polar-Polar | 89 | 119 | 30 |
