# Supplementary material for: Next generation sequencing reveals the antibiotic resistant variants in the genome of Pseudomonas aeruginosa
Source: PLoS One. 2017 Aug 10;12(8):e0182524. doi: 10.1371/journal.pone.0182524 (PMC5557631; doi:10.1371/journal.pone.0182524)
Supplement: S4 Table — The Colistin susceptible isolates PAS 3, 4, 5 and 8 were compared against the rest of the resistant isolates. (DOCX) [file pone.0182524.s004.docx]

**S4 Table. Non-synonymous SNP’s in Colistin resistant isolates.** The Colistin susceptible isolates PAS 3, 4, 5 and 8 were compared against the rest of the resistant isolates

| S. No | **Nucleotide Position** | **Susceptible genome** | **Alteration** | **Gene ID** | **AA changes** | **Hydrophobicity** | **Charges** | **Polarity** | **Sequence length in reference** | **Sequence length in isolate** | **Nucleotide difference** |
| --- | --- | --- | --- | --- | --- | --- | --- | --- | --- | --- | --- |
| 1 | 23107 | T | C | PA0021 | L79P | hydrophobic-hydrophobic | neutral-neutral | Non-polar-Non-polar | 131 | 115 | 16 |
| 2 | 49850 | A | G | PA0041 | T2313A | hydrophilic-hydrophobic | neutral-neutral | Polar-Non-polar | 119 | 89 | 30 |
| 3 | 173411 | G | C | PA0151 | V789L | hydrophobic-hydrophobic | neutral-neutral | Non-polar-Non-polar | 117 | 131 | 14 |
| 4 | 187205 | G | C | PA0164 | G125A | hydrophobic-hydrophobic | neutral-neutral | Non-polar-Non-polar | 75 | 89 | 14 |
| 5 | 196376 | G | T | PA0172 | R125S | hydrophilic-hydrophilic | positive-neutral | Polar-Polar | 174 | 105 | 69 |
| 6 | 196398 | G | C | PA0172 | H117Q | hydrophilic-hydrophilic | positive-neutral | Polar-Polar | 155 | 146 | 9 |
| 7 | 584592 | A | G | PA0525 | N246D | hydrophilic-hydrophilic | neutral-negative | Polar-Polar | 132 | 133 | 1 |
| 8 | 688201 | G | A | PA0629 | G75S | hydrophobic-hydrophilic | neutral-neutral | Non-polar-Polar | 75 | 105 | 30 |
| 9 | 736844 | C | A | PA0677 | S85I | hydrophilic-hydrophobic | neutral-neutral | Polar-Non-polar | 105 | 131 | 26 |
| 10 | 766219 | G | C | exbB2 | G302A | hydrophobic-hydrophobic | neutral-neutral | Non-polar-Non-polar | 75 | 89 | 14 |
| 11 | 825396 | T | G | PA0757 | D108E | hydrophilic-hydrophilic | negative-negative | Polar-Polar | 133 | 147 | 14 |
| 12 | 879087 | T | A | PA0800 | W21R | hydrophilic-hydrophilic | neutral-positive | Polar-Polar | 204 | 174 | 30 |
| 13 | 879094 | A | C | PA0800 | D23A | hydrophilic-hydrophobic | negative-neutral | Polar-Non-polar | 133 | 89 | 44 |
| 14 | 1102874 | T | C | pauA | F649L | hydrophobic-hydrophobic | neutral-neutral | Non-polar-Non-polar | 165 | 131 | 34 |
| 15 | 1230976 | G | A | PA1139 | V223I | hydrophobic-hydrophobic | neutral-neutral | Non-polar-Non-polar | 117 | 131 | 14 |
| 16 | 1326531 | T | C | PA1223 | T137A | hydrophilic-hydrophobic | neutral-neutral | Polar-Non-polar | 119 | 89 | 30 |
| 17 | 1366579 | A | C | PA1259 | S568A | hydrophilic-hydrophobic | neutral-neutral | Polar-Non-polar | 105 | 89 | 16 |
| 18 | 1373185 | T | C | PA1264 | T221A | hydrophilic-hydrophobic | neutral-neutral | Polar-Non-polar | 119 | 89 | 30 |
| 19 | 1377249 | G | C | PA1267 | A65G | hydrophobic-hydrophobic | neutral-neutral | Non-polar-Non-polar | 89 | 75 | 14 |
| 20 | 1386984 | A | G | cobD | D221G | hydrophilic-hydrophobic | negative-neutral | Polar-Non-polar | 133 | 75 | 58 |
| 21 | 1387086 | T | C | cobD | F255S | hydrophobic-hydrophilic | neutral-neutral | Non-polar-Polar | 165 | 105 | 60 |
| 22 | 1465853 | G | A | PA1351 | V326I | hydrophobic-hydrophobic | neutral-neutral | Non-polar-Non-polar | 117 | 131 | 14 |
| 23 | 1550472 | G | A | PA1424 | S197N | hydrophilic-hydrophilic | neutral-neutral | Polar-Polar | 105 | 132 | 27 |
| 24 | 1551303 | C | A | PA1425 | A107D | hydrophobic-hydrophilic | neutral-negative | Non-polar-Polar | 89 | 133 | 44 |
| 25 | 1707135 | C | G | PA1566 | E134D | hydrophilic-hydrophilic | negative-negative | Polar-Polar | 147 | 133 | 14 |
| 26 | 1844667 | A | G | pscP | V196A | hydrophobic-hydrophobic | neutral-neutral | Non-polar-Non-polar | 117 | 89 | 28 |
| 27 | 1856415 | G | C | exsE | E36D | hydrophilic-hydrophilic | negative-negative | Polar-Polar | 147 | 133 | 14 |
| 28 | 1876131 | C | A | PA1733 | L95M | hydrophobic-hydrophobic | neutral-neutral | Non-polar-Non-polar | 131 | 149 | 18 |
| 29 | 2099646 | G | C | PA1923 | E65D | hydrophilic-hydrophilic | negative-negative | Polar-Polar | 147 | 133 | 14 |
| 30 | 2099662 | A | G | PA1923 | N71D | hydrophilic-hydrophilic | neutral-negative | Polar-Polar | 132 | 133 | 1 |
| 31 | 2099713 | G | C | PA1923 | E88Q | hydrophilic-hydrophilic | negative-neutral | Polar-Polar | 147 | 146 | 1 |
| 32 | 2141175 | T | C | PA1956 | S105G | hydrophilic-hydrophobic | neutral-neutral | Polar-Non-polar | 105 | 75 | 30 |
| 33 | 2157492 | A | G | pqqF | K707E | hydrophilic-hydrophilic | positive-negative | Polar-Polar | 146 | 147 | 1 |
| 34 | 2194717 | C | G | PA2006 | E232Q | hydrophilic-hydrophilic | negative-neutral | Polar-Polar | 147 | 146 | 1 |
| 35 | 2250038 | T | C | PA2056 | C116R | hydrophobic-hydrophilic | neutral-positive | Non-polar-Polar | 121 | 174 | 53 |
| 36 | 2309992 | G | A | PA2098 | G184S | hydrophobic-hydrophilic | neutral-neutral | Non-polar-Polar | 75 | 105 | 30 |
| 37 | 2322502 | T | C | PA2111 | I94V | hydrophobic-hydrophobic | neutral-neutral | Non-polar-Non-polar | 131 | 117 | 14 |
| 38 | 2341243 | G | C | PA2127 | A133G | hydrophobic-hydrophobic | neutral-neutral | Non-polar-Non-polar | 89 | 75 | 14 |
| 39 | 2384930 | C | T | PA2163 | R558Q | hydrophilic-hydrophilic | positive-neutral | Polar-Polar | 174 | 146 | 28 |
| 40 | 2400663 | A | G | PA2179 | S315P | hydrophilic-hydrophobic | neutral-neutral | Polar-Non-polar | 105 | 115 | 10 |
| 41 | 2523932 | A | C | PA2293 | D90E | hydrophilic-hydrophilic | negative-negative | Polar-Polar | 133 | 147 | 14 |
| 42 | 2536616 | C | G | ambE | V810L | hydrophobic-hydrophobic | neutral-neutral | Non-polar-Non-polar | 117 | 131 | 14 |
| 43 | 2541109 | C | G | ambC | S20T | hydrophilic-hydrophilic | neutral-neutral | Polar-Polar | 105 | 119 | 14 |
| 44 | 2541237 | A | G | ambB | I1237T | hydrophobic-hydrophilic | neutral-neutral | Non-polar-Polar | 131 | 119 | 12 |
| 45 | 2565208 | T | C | PA2324 | C307R | hydrophobic-hydrophilic | neutral-positive | Non-polar-Polar | 121 | 174 | 53 |
| 46 | 2607437 | A | G | PA2360 | S266P | hydrophilic-hydrophobic | neutral-neutral | Polar-Non-polar | 105 | 115 | 10 |
| 47 | 2609544 | A | G | PA2361 | L834P | hydrophobic-hydrophobic | neutral-neutral | Non-polar-Non-polar | 131 | 115 | 16 |
| 48 | 2609545 | G | C | PA2361 | L834V | hydrophobic-hydrophobic | neutral-neutral | Non-polar-Non-polar | 131 | 117 | 14 |
| 49 | 2611137 | A | G | PA2361 | V303A | hydrophobic-hydrophobic | neutral-neutral | Non-polar-Non-polar | 117 | 89 | 28 |
| 50 | 2866467 | T | C | PA2539 | E347G | hydrophilic-hydrophobic | negative-neutral | Polar-Non-polar | 147 | 75 | 72 |
| 51 | 3002489 | T | C | PA2653 | N237S | hydrophilic-hydrophilic | neutral-neutral | Polar-Polar | 132 | 105 | 27 |
| 52 | 3071207 | C | T | PA2716 | V250I | hydrophobic-hydrophobic | neutral-neutral | Non-polar-Non-polar | 117 | 131 | 14 |
| 53 | 3175763 | T | G | PA2822 | I120L | hydrophobic-hydrophobic | neutral-neutral | Non-polar-Non-polar | 131 | 131 | 0 |
| 54 | 3179624 | T | C | ospR | T113A | hydrophilic-hydrophobic | neutral-neutral | Polar-Non-polar | 119 | 89 | 30 |
| 55 | 3186491 | C | A | PA2834 | L15M | hydrophobic-hydrophobic | neutral-neutral | Non-polar-Non-polar | 131 | 149 | 18 |
| 56 | 3221683 | T | C | PA2869 | L11P | hydrophobic-hydrophobic | neutral-neutral | Non-polar-Non-polar | 131 | 115 | 16 |
| 57 | 3262164 | G | A | cobL | A215T | hydrophobic-hydrophilic | neutral-neutral | Non-polar-Polar | 89 | 119 | 30 |
| 58 | 3284255 | T | C | PA2928 | N115S | hydrophilic-hydrophilic | neutral-neutral | Polar-Polar | 132 | 105 | 27 |
| 59 | 3474074 | T | C | PA3093 | S353P | hydrophilic-hydrophobic | neutral-neutral | Polar-Non-polar | 105 | 115 | 10 |
| 60 | 3486431 | T | C | PA3106 | I252V | hydrophobic-hydrophobic | neutral-neutral | Non-polar-Non-polar | 131 | 117 | 14 |
| 61 | 3684683 | G | T | PA3291 | T12N | hydrophilic-hydrophilic | neutral-neutral | Polar-Polar | 119 | 132 | 13 |
| 62 | 3684701 | C | T | PA3291 | S6N | hydrophilic-hydrophilic | neutral-neutral | Polar-Polar | 105 | 132 | 27 |
| 63 | 3684972 | A | T | PA3292 | V264D | hydrophobic-hydrophilic | neutral-negative | Non-polar-Polar | 117 | 133 | 16 |
| 64 | 3685006 | A | G | PA3292 | S253P | hydrophilic-hydrophobic | neutral-neutral | Polar-Non-polar | 105 | 115 | 10 |
| 65 | 3685159 | A | G | PA3292 | S202P | hydrophilic-hydrophobic | neutral-neutral | Polar-Non-polar | 105 | 115 | 10 |
| 66 | 3685215 | T | A | PA3292 | D183V | hydrophilic-hydrophobic | negative-neutral | Polar-Non-polar | 133 | 117 | 16 |
| 67 | 3685287 | A | T | PA3292 | V159D | hydrophobic-hydrophilic | neutral-negative | Non-polar-Polar | 117 | 133 | 16 |
| 68 | 3685386 | T | C | PA3292 | Y126C | hydrophilic-hydrophobic | neutral-neutral | Polar-Non-polar | 181 | 121 | 60 |
| 69 | 3704096 | T | C | PA3305 | T356A | hydrophilic-hydrophobic | neutral-neutral | Polar-Non-polar | 119 | 89 | 30 |
| 70 | 3704374 | C | T | PA3305 | R263Q | hydrophilic-hydrophilic | positive-neutral | Polar-Polar | 174 | 146 | 28 |
| 71 | 3818184 | A | C | PA3409 | S117A | hydrophilic-hydrophobic | neutral-neutral | Polar-Non-polar | 105 | 89 | 16 |
| 72 | 3820723 | T | C | PA3414 | V139A | hydrophobic-hydrophobic | neutral-neutral | Non-polar-Non-polar | 117 | 89 | 28 |
| 73 | 3910588 | T | C | PA3491 | I726T | hydrophobic-hydrophilic | neutral-neutral | Non-polar-Polar | 131 | 119 | 12 |
| 74 | 3971730 | T | G | algX | Y49D | hydrophilic-hydrophilic | neutral-negative | Polar-Polar | 181 | 133 | 48 |
| 75 | 4167279 | C | A | PA3722 | A82S | hydrophobic-hydrophilic | neutral-neutral | Non-polar-Polar | 89 | 105 | 16 |
| 76 | 4334711 | A | C | PA3871 | S171A | hydrophilic-hydrophobic | neutral-neutral | Polar-Non-polar | 105 | 89 | 16 |
| 77 | 4500983 | G | C | PA4021 | A317G | hydrophobic-hydrophobic | neutral-neutral | Non-polar-Non-polar | 89 | 75 | 14 |
| 78 | 4522521 | A | G | PA4039 | D527G | hydrophilic-hydrophobic | negative-neutral | Polar-Non-polar | 133 | 75 | 58 |
| 79 | 4671622 | A | G | piv | T102A | hydrophilic-hydrophobic | neutral-neutral | Polar-Non-polar | 119 | 89 | 30 |
| 80 | 4694385 | T | C | bfiS | T551A | hydrophilic-hydrophobic | neutral-neutral | Polar-Non-polar | 119 | 89 | 30 |
| 81 | 4823319 | T | G | PA4298 | T16P | hydrophilic-hydrophobic | neutral-neutral | Polar-Non-polar | 119 | 115 | 4 |
| 82 | 4849311 | A | G | PA4320 | T280A | hydrophilic-hydrophobic | neutral-neutral | Polar-Non-polar | 119 | 89 | 30 |
| 83 | 5085360 | G | C | PA4541 | S973T | hydrophilic-hydrophilic | neutral-neutral | Polar-Polar | 105 | 119 | 14 |
| 84 | 5491479 | C | T | PA4897 | A45V | hydrophobic-hydrophobic | neutral-neutral | Non-polar-Non-polar | 89 | 117 | 28 |
| 85 | 5554373 | A | C | PA4949 | S236R | hydrophilic-hydrophilic | neutral-positive | Polar-Polar | 105 | 174 | 69 |
| 86 | 5570022 | A | C | PA4961 | E311D | hydrophilic-hydrophilic | negative-negative | Polar-Polar | 147 | 133 | 14 |
| 87 | 5761034 | G | A | PA5114 | P104S | hydrophobic-hydrophilic | neutral-neutral | Non-polar-Polar | 115 | 105 | 10 |
| 88 | 5859820 | A | G | PA5205 | I10V | hydrophobic-hydrophobic | neutral-neutral | Non-polar-Non-polar | 131 | 117 | 14 |
| 89 | 5877231 | G | T | PA5219 | A190S | hydrophobic-hydrophilic | neutral-neutral | Non-polar-Polar | 89 | 105 | 16 |
| 90 | 5965829 | C | T | poxB | A324V | hydrophobic-hydrophobic | neutral-neutral | Non-polar-Non-polar | 89 | 117 | 28 |
| 91 | 5978437 | A | C | PA5309 | T217P | hydrophilic-hydrophobic | neutral-neutral | Polar-Non-polar | 119 | 115 | 4 |
| 92 | 6236006 | T | C | PA5543 | H75R | hydrophilic-hydrophilic | positive-positive | Polar-Polar | 155 | 174 | 19 |
| 93 | 6245826 | C | T | PA5551 | D163N | hydrophilic-hydrophilic | negative-neutral | Polar-Polar | 133 | 132 | 1 |
