# Supplementary material for: Next generation sequencing reveals the antibiotic resistant variants in the genome of Pseudomonas aeruginosa
Source: PLoS One. 2017 Aug 10;12(8):e0182524. doi: 10.1371/journal.pone.0182524 (PMC5557631; doi:10.1371/journal.pone.0182524)
Supplement: S5 Table — The Tazobactam susceptible isolate PAS 7 was compared against the rest of the resistant isolates. (DOCX) [file pone.0182524.s005.docx]

**S5 Table. Non-synonymous SNP’s in Piperacillin/Tazobactam resistant isolates.** The Tazobactam susceptible isolate PAS 7 was compared against the rest of the resistant isolates.

| S. No | **Nucleotide Position** | **Susceptible genome** | **Alteration** | **Gene ID** | **AA changes** | **Hydrophobicity** | **Charges** | **Polarity** | **Sequence length in reference** | **Sequence length in isolate** | **Nucleotide difference** |
| --- | --- | --- | --- | --- | --- | --- | --- | --- | --- | --- | --- |
| 1 | 634110 | T | A | PA0575 | Y52F | hydrophilic-hydrophobic | neutral-neutral | Polar-Non-polar | 181 | 165 | 16 |
| 2 | 712782 | C | T | PA0659 | V126I | hydrophobic-hydrophobic | neutral-neutral | Non-polar-Non-polar | 117 | 131 | 14 |
| 3 | 2611495 | T | G | PA2361 | M184L | hydrophobic-hydrophobic | neutral-neutral | Non-polar-Non-polar | 149 | 131 | 18 |
| 4 | 3253437 | C | T | PA2898 | A82T | hydrophobic-hydrophilic | neutral-neutral | Non-polar-Polar | 89 | 119 | 30 |
| 5 | 3724720 | T | G | PA3322 | Q145H | hydrophilic-hydrophilic | neutral-positive | Polar-Polar | 146 | 155 | 9 |
| 6 | 4522427 | C | A | PA4039 | L496M | hydrophobic-hydrophobic | neutral-neutral | Non-polar-Non-polar | 131 | 149 | 18 |
